# Crystal structure and Hirshfeld surface analysis of (*Z*)-3-methyl-4-(thio­phen-2-yl­methyl­idene)isoxazol-5(4*H*)-one

**DOI:** 10.1107/S2056989021002632

**Published:** 2021-03-12

**Authors:** Rima Laroum, Assia Benouatas, Noudjoud Hamdouni, Wissame Zemamouche, Ali Boudjada, Abdelmadjid Debache

**Affiliations:** a Laboratoire de Synthèse de Molécules d’Intérêts Biologiques, Département de Chimie, Université Mentouri Constantine, 25000, Algeria; b Laboratoire de Cristallographie, Département de Physique, Université Mentouri Constantine, 25000, Algeria

**Keywords:** crystal structure, π–π inter­actions, isoxazole, Hirshfeld surface

## Abstract

The title compound, C_9_H_7_NO_2_S crystallizes with two independent mol­ecules (*A* and *B*) in the asymmetric unit with *Z* = 8. Both mol­ecules are almost planar with a dihedral angle between the isoxazole and thio­phen rings of 3.67 (2)° in mol­ecule *A* and 10.00 (1) ° in mol­ecule *B*. The packing of mol­ecules *A* and *B* is of an *ABAB⋯* type along the *b*-axis direction, the configuration about the C=C bond is *Z*. In the crystal, the presence of C—H⋯O, C—H⋯ N and π–π inter­actions [centroid–centroid distances of 3.701 (2) and 3.766 (2) Å] link the mol­ecules into a three-dimensional architecture. An analysis of Hirshfeld surfaces shows the importance of C—H⋯O and C—H⋯N hydrogen bonds in the packing mechanism of the crystalline structure.

## Abstract 2

The title compound, C_9_H_7_NO_2_S, crystallizes with two independent mol­ecules (*A* and *B*) in the asymmetric unit with *Z* = 4.In the mol­ecular skeleton of title compound, the angle between mean planes of the two mol­ecules *A* and *B* is 4.09 (1)°. The two mol­ecules *A* and *B* are involved in inter­molecular C—H⋯O and C—H⋯N hydrogen bonds.

## Chemical context   

Isoxazolones show some inter­esting biological properties. They are inhibitors of the factorization of tumor necrosis alpha (TNF-α) (Laughlin *et al.*, 2005 ▸) and anti­microbial (Mazimba *et al.*, 2014 ▸). They are used for the treatment of cerebrovascular disorders and as muscle relaxants. They are also herbicides (Tomita *et al.*, 1977 ▸) and fungicides (Miyake *et al.*, 2012 ▸). On other hand, isoxazolone derivatives constitute excellent inter­mediates for the synthesis of various heterocycles such as pyrido­pyrimidines (Tu *et al.*, 2006 ▸), quinolines (Abbiati *et al.*, 2003 ▸) and undergo various chemical transformations (Batra & Bhaduri, 1994 ▸). Some cyclo­addition reactions are also described and provide access to several types of polycycles (Badrey & Gomha, 2014 ▸). For these reasons, these compounds have been the subject of several investigations. The present method for their synthesis is a three-component polycondensation between an aromatic aldehyde, ethyl aceto­acetate and hydroxyl­amine hydro­chloride under different conditions and for our part we propose here the use of K_2_CO_3_, a food additive, tolerated in organic agriculture, very inexpensive, highly available and a safe catalyst, in an aqueous medium. In the present study, we report on the synthesis, mol­ecular and crystal structure together with a Hirshfeld surface analysis of the title isoxazole derivative.

## Structural commentary   

**Figure 1:**
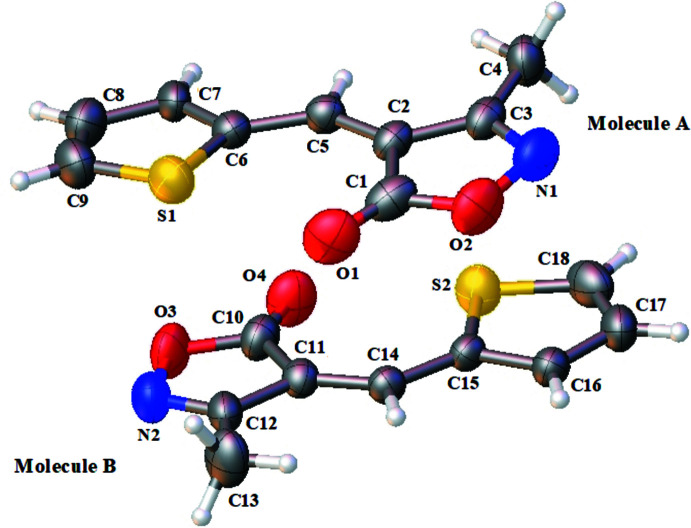
The mol­ecular structure of the title compound, with atom labelling and displacement ellipsoids drawn at the 50% probability level.

The mol­ecular structure of the title compound is shown in (Fig. 1 ▸). It crystallizes with two independent mol­ecules (*A* and *B*) in the asymmetric unit. The mol­ecular structure adopts a *Z*-configuration about the C=C [1.354 (3) Å in mol­ecule *A* and 1.357 (3) Å in mol­ecule *B*] double bonds. 
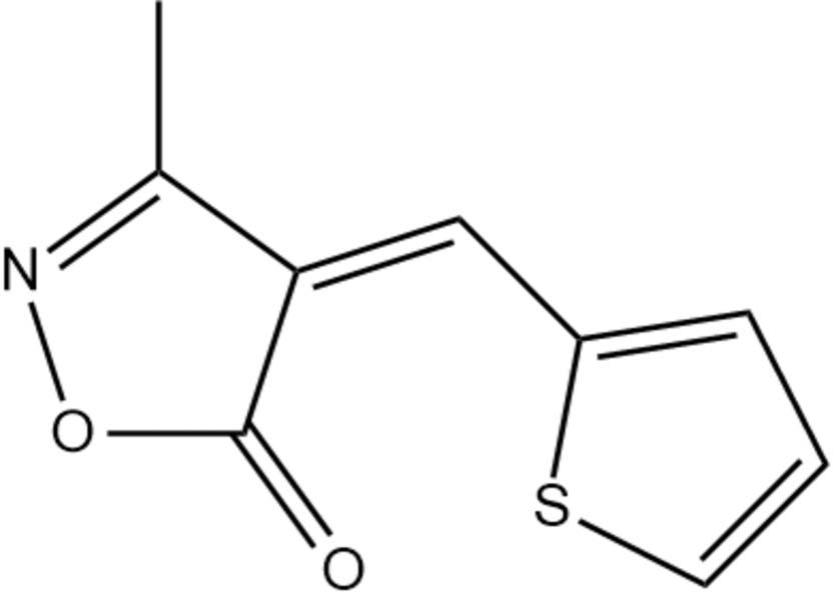


The bond lengths in the two mol­ecules are practically equal, while there are slight differences in bond angles; with for example C2—C3—C4 (mol­ecule *A*) and C11—C12—C13 (mol­ecule *B*) differing by 0.8 (2)°. Also, a slight difference of 0.3 (2)° is observed between the angles C2—C5—C6 and C11—C14—C15. In mol­ecule *A*, the angle between the normal of the mol­ecular plane (O2*A*/N1*A*/C1*A*–C3*A*) and the normal of the (S1*A*/C6*A*–C9*A*) plane is 3.67 (2)°. An important difference is observed in mol­ecule *B*, where the angle between the normal of the mol­ecular plane (O3*B*/N2*B*/C10*B*–C12*B*) and the normal of the (S2*B*/C15*B*–C18*B*) plane is 10.00 (1)°. In the mol­ecular skeleton, the angle between the mean planes of the mol­ecules *A* and *B* is 4.09 (1)°. Each of the two methyl groups, C4 and C13, has a C—H bond lying in the mean plane of the mol­ecular skeleton, and they are oriented toward the thio­phene group.

## Supra­molecular features   

**Figure 2:**
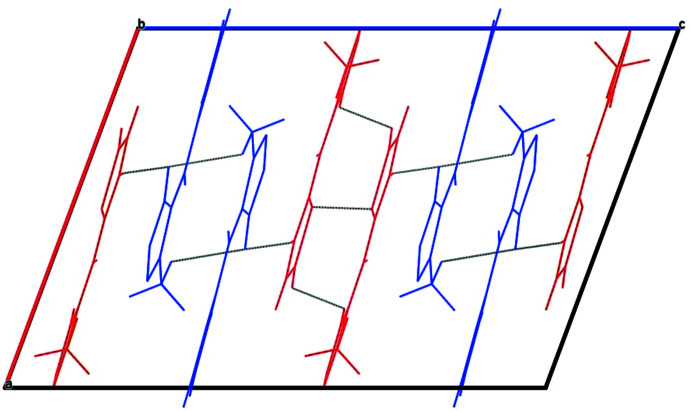
A view along the *b* axis of the crystal packing of the title compound (mol­ecule *A* in blue and mol­ecule *B* in red).

In the crystal, the structure consists of wavy layers containing mol­ecules of the same type, forming an alternated packing described by an *ABAB*⋯ sequence (Fig. 2 ▸). The mol­ecules form infinite chains along the *b*-axis direction. They are linked by offset π–π inter­actions: [*Cg*1⋯*Cg*2^i^ = 3.701 (2) Å and *Cg*3⋯*Cg*4^ii^ = 3.766 (2) Å where *Cg*1, *Cg*2, *Cg*3 and *Cg*4 are the centroids of the O2*A*/N1*A*/C1*A*–C3A, S2*B*/C15*B*–C18*B*, S1A/C6*A*–C9*A* and O3*B*/N2*B*/C10*B*–C12*B* rings, respectively; symmetry codes: (i) −*x*, 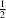 + *y*, 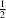 − *z*; (ii) −*x*, 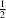 − *y*, 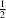 + *z*]. The two mol­ecules *A* and *B* are involved in inter­molecular C—H⋯O and C—H⋯N hydrogen bonds (Table 1 ▸).

## Analysis of the Hirshfeld surfaces   

**Figure 3:**
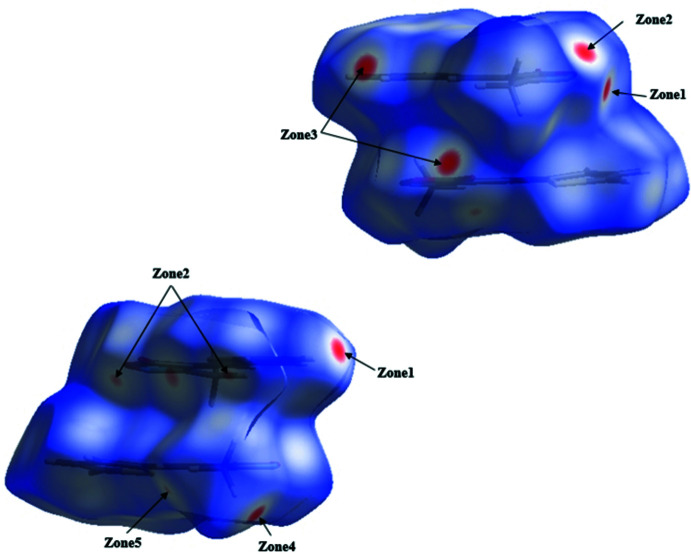
Two views of the Hirshfeld surface mapped over *d*_norm_.

**Figure 4:**
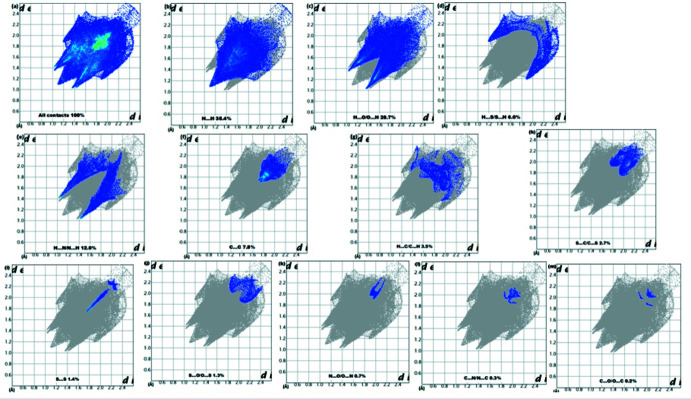
Two-dimensional finger print plots: (*a*) overall, and delineated into contributions from different contacts: (*b*) H⋯H, (*c*) H⋯O/O⋯H, (*d*) H⋯S/S⋯H, (*e*) H⋯N/N⋯H and (*f*) C⋯C.

The Hirshfeld surface analysis (Spackman & Jayatilaka, 2009 ▸) and the associated two-dimensional fingerprint plots (McKinnon *et al.*, 2007 ▸) were generated with *CrystalExplorer* (Turner *et al.*, 2017 ▸). The analysis of Hirshfeld surface mapped over *d*_norm_ is shown in (Fig. 3 ▸). The inter­actions between the corresponding donor and acceptor atoms are visualized as bright-red spots on both sides (zones 1, 2, 3 and 4) of the Hirshfeld surfaces (Fig. 3 ▸), corresponding to C17—H17⋯N2, C4—H4*C*⋯N2, C16—H16⋯O2 and C18—H18⋯O4 hydrogen bonds, respectively. Two other red spots exist, corresponding to C4—H4*A*⋯O inter­actions (Fig. 3 ▸, zone 5), are considered to be very weak inter­actions, comparing them to the van der Waals radii. The overall two-dimensional fingerprint plot of the structure and H⋯S/S⋯H, H⋯H, H⋯O/O⋯H, H⋯N/N⋯H and C⋯C contacts are illus­trated in Fig. 4 ▸ *a–m*). The H⋯H contacts, accounting for about 35.4% of the Hirshfeld surface (Fig. 4 ▸ *b*) represent the largest contribution and are seen in the fingerprint plot as a pair of shorts pikes at *d*_e_ + *d*_i_ = 2.2 Å; comparing this to van der Waals radius, we find the difference between them is about 1 Å, which means it is a very powerful inter­action. H⋯O/O⋯H contacts (Fig. 4 ▸ *c*) make a contribution of 28.7%, with a distinctive peak in the fingerprint plot at *d*_e_ + *d*_i_ = 2.4 Å; the van der Waals radius sum for this inter­action is about 2.7 Å.

**Figure 5:**
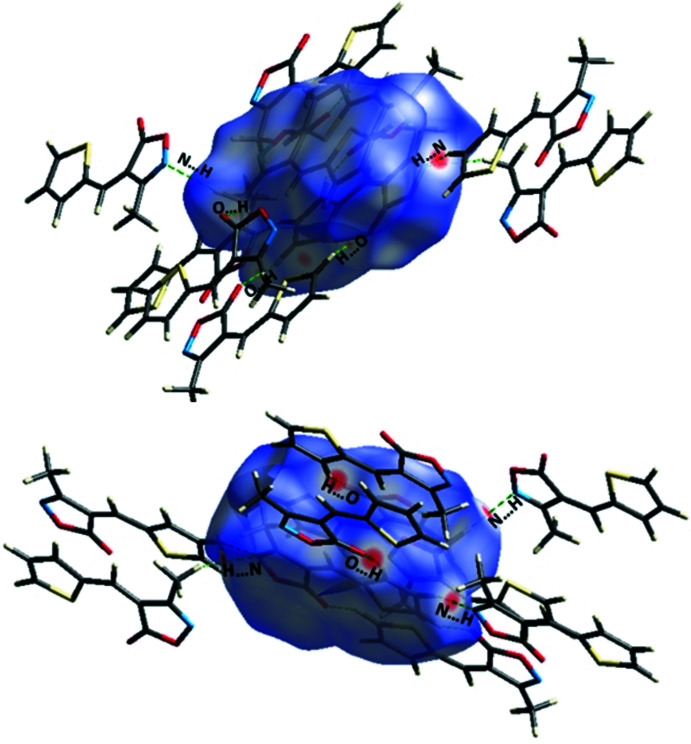
Two views of the Hirshfeld surface mapped over *d*_norm_, with inter­actions to neighbouring mol­ecules shown as green dashed lines.

The pair of short peaks at *d*_e_ + *d*_i_ = 3.1, *i.e*. almost equal to the sum of the van der Waals radius, in the fingerprint plot delineated into H⋯S/S⋯H contacts are indicative of short inter­atomic contacts in the crystal (6% contribution, Fig. 4 ▸ *d*). Although the H⋯N /N⋯H inter­actions have a notable contribution of 12% to the Hirshfeld surface (Fig. 4 ▸ *e*), their inter­atomic distances (*d*_e_ + *d*_i_ = 2.4 Å) are less than their van der Waals radius (2.7 Å), which means that it is a very strong inter­action in this structure. The presence of π–π stacking reflects the presence of C⋯C contacts (Fig. 4 ▸ *f*), which account for 7.9% of the Hirshfeld surface with *d*_e_ + *d*_i_ = 3.4 Å; the van der Waals radius is 3.4 Å, so we can confirm the presence of π–π stacking. Two further views of the Hirshfeld surface are shown in Fig. 5 ▸.

## Database survey   

A search of the Cambridge Structural Database (CSD, v5.40, last update May 2019; Groom *et al.*, 2016 ▸) for the (*Z*)-4-(thio­phen-2-yl­methyl­idene)isoxazol-5(4*H*)-one unit gave five hits: 4-(2-hydroxybenzyl­idene)-3-methyl­isoxazol-5(4*H*)-one (AJESAK; Cheng *et al.*, 2009 ▸), 2-(naphthalen-1-yl)-4-(thio­phen-2-yl­methyl­idene)-1,3-oxazol-5(4*H*)-one (ERIXIN; Gündoğdu *et al.*, 2011 ▸), (*Z*)-4-benzyl­idene-3-methyl­isoxazol-5(4*H*)-one (MBYIOZ01; Chandra *et al.*, 2012 ▸), 2-methyl-4-(thio­phen-2-yl­methyl­idene)-1,3-oxazol-5(4*H*)-one (WOYPIL; Sharma *et al.*, 2015 ▸) and (*Z*)-4-(4-hy­droxy­benzyl­idene)-3-methyl­isoxazol-5(4*H*)-one (VIDSAF; Zemamouche *et al.*, 2018 ▸).

The asymmetric unit of the title compound contains two crystallographically independent mol­ecules, as found for ERIXIN and WOYPIL while in AJESAK, MBYIOZ01 and VIDSAF, there is only one mol­ecule per asymmetric unit. The configuration about the C=C bond is *Z* in all five compounds and in each mol­ecule, the oxazol and thio­phene rings are inclined to one another by 3.67 (2), 10.00 (1), 0.86 (9), 7.02 (8), 2.65 (16), 4.55 (15), 6.50 (1), 7.98 (8) and 3.18 (8)°, respectively.

In the crystal of WOYPIL, the individual mol­ecules are linked *via* C—H⋯O hydrogen bonds, forming *ABAB* chains along the [10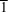] direction, similarly in the crystal of the title compound, the packing of mol­ecules *A* and *B* is of an *ABAB*⋯ type along the [100] direction. In our compound, the cohesion of the crystal is ensured by inter­actions of the type C—H⋯O, C—H⋯π and π–π [inter­centroid distances of 3.701 (2) and 3.766 (2) Å compared with 3.811 (2) and 3.889 (2) Å in ERIXIN and 3.767 (2) and 3.867 (2) Å in WOYPIL].

## Synthesis and crystallization   

Thio­phene-2-carbaldehyde (C_5_H_4_OS, 1 mmol), hydroxyl­amine hydro­chloride (ClH_4_NO, 1 mmol), ethyl aceto­acetate (C_6_H_10_O_3_,1 mmol) and K_2_CO_3_ (5 mol%) were mixed in a 25 mL flask equipped with a magnetic stirrer. The mixture was refluxed in 5 mL of water for 3h (followed by TLC). When the reaction was judged to be finished, the mixture was gradually poured into ice-cold water. Stirring was maintained for a few minutes and the obtained solid was filtered and purified by crystallization from ethanol (yield 72%).

## Refinement details   

Crystal data, data collection and structure refinement details for the title compound are summarized in Table 2 ▸. H atoms were placed in calculated positions (C—H = 0.93–0.96 Å) and refined as riding with *U*_iso_(H) = 1.2–1.5*U*_eq_(C).

## Supplementary Material

Crystal structure: contains datablock(s) I. DOI: 10.1107/S2056989021002632/zn2002sup1.cif

Structure factors: contains datablock(s) I. DOI: 10.1107/S2056989021002632/zn2002Isup2.hkl

Supporting information file. DOI: 10.1107/S2056989021002632/zn2002Isup3.cml

CCDC reference: 2069004

Additional supporting information:  crystallographic information; 3D view; checkCIF report

## Figures and Tables

**Table 1:** Hydrogen-bond geometry (Å, °)

*D*—H⋯*A*	*D*—H	H⋯*A*	*D*⋯*A*	*D*—H⋯*A*
C7—H7⋯O4^i^	0.93	2.51	3.387 (3)	156
C8—H8⋯N1^ii^	0.93	2.58	3.491 (5)	166
C13—H13c⋯N1^iii^	0.96	2.57	3.487 (4)	160

Symmetry codes: (i) -x+1, -y+1, -z+1; (ii) x-1, y, z; (iii) x-1, -y+{\script{1\over 2}}, z-{\script{1\over 2}}.

**Table 2:** Experimental details

Crystal data	
Chemical formula	C_9_H_7_NO_2_S
*M*_r_	193.22
Crystal system, space group	Monoclinic, *P*2_1_/*c*
Temperature (K)	301
*a*, *b*, *c* (Å)	10.4660 (4), 12.1614 (5), 14.7636 (6)
β (°)	110.362 (1)
*V* (Å^3^)	1761.71 (12)
*Z*	8
Radiation type	Mo *K*α
μ (mm^−1^)	0.33
Crystal size (mm)	0.31 × 0.20 × 0.10

Data collection	
Diffractometer	Agilent Technologies Xcalibur, Eos
Absorption correction	Multi-scan (*CrysAlis PRO*; Agilent, 2013 ▸))
*T*_min_, *T*_max_	0.758, 0.968
No. of measured, independent and observed [*I* > 2σ(*I*)] reflections	72470, 6743, 4110
*R*_int_	0.084
(sin θ/λ)_max_ (Å^−1^)	0.770

Refinement	
*R*[*F* ^2^ > 2σ(*F* ^2^)], *wR*(*F* ^2^), *S*	0.079, 0.239, 1.07
No. of reflections	6743
No. of parameters	235
H-atom treatment	H-atom parameters constrained
Δρ_max_, Δρ_min_ (e Å^−3^)	0.60, −0.54

Computer programs: *CrysAlis PRO* (Agilent, 2013 ▸), *SHELXT* (Sheldrick, 2015*a* ▸), *SHELXL2018/3* (Sheldrick, 2015*b* ▸), *ORTEP-3 for Windows* and *WinGX* publication routines (Farrugia, 2012 ▸) and *Mercury* (Macrae *et al.*, 2020 ▸).

## supplementary crystallographic information

### Crystal data

**Table:**

C_9_H_7_NO_2_S	*F*(000) = 800
*M**_r_* = 193.22	*D*_x_ = 1.457 Mg m^−^^3^
Monoclinic, *P*2_1_/*c*	Mo *K*α radiation, λ = 0.71073 Å
Hall symbol: -P 2ybc	Cell parameters from 6745 reflections
*a* = 10.4660 (4) Å	θ = 2.2–33.2°
*b* = 12.1614 (5) Å	µ = 0.33 mm^−^^1^
*c* = 14.7636 (6) Å	*T* = 301 K
β = 110.362 (1)°	Needle, white
*V* = 1761.71 (12) Å^3^	0.31 × 0.20 × 0.10 mm
*Z* = 8	

### Data collection

**Table:**

Agilent Technologies Xcalibur, Eos diffractometer	6743 independent reflections
Radiation source: Enhance (Mo) X-ray Source	4110 reflections with *I* > 2σ(*I*)
Detector resolution: 8.02 pixels mm^-1^	*R*_int_ = 0.084
ω scans	θ_max_ = 33.2°, θ_min_ = 2.2°
Absorption correction: multi-scan (CrysAlis PRO; Agilent, 2013))	*h* = −16→16
*T*_min_ = 0.758, *T*_max_ = 0.968	*k* = −18→18
72470 measured reflections	*l* = −22→22

### Refinement

**Table:**

Refinement on *F*^2^	Primary atom site location: structure-invariant direct methods
Least-squares matrix: full	Secondary atom site location: difference Fourier map
*R*[*F*^2^ > 2σ(*F*^2^)] = 0.079	Hydrogen site location: inferred from neighbouring sites
*wR*(*F*^2^) = 0.239	H-atom parameters constrained
*S* = 1.07	*w* = 1/[σ^2^(*F*_o_^2^) + (0.1035*P*)^2^ + 1.1548*P*] where *P* = (*F*_o_^2^ + 2*F*_c_^2^)/3
6743 reflections	(Δ/σ)_max_ < 0.001
235 parameters	Δρ_max_ = 0.60 e Å^−^^3^
0 restraints	Δρ_min_ = −0.54 e Å^−^^3^
0 constraints	

### Special details

**Table:**

Geometry. All esds (except the esd in the dihedral angle between two l.s. planes) are estimated using the full covariance matrix. The cell esds are taken into account individually in the estimation of esds in distances, angles and torsion angles; correlations between esds in cell parameters are only used when they are defined by crystal symmetry. An approximate (isotropic) treatment of cell esds is used for estimating esds involving l.s. planes.

### Fractional atomic coordinates and isotropic or equivalent isotropic displacement parameters (Å^2^)

**Table:**

	*x*	*y*	*z*	*U*_iso_*/*U*_eq_	
S1	0.50289 (8)	0.12735 (6)	0.55602 (6)	0.0497 (2)	
S2	0.83062 (8)	0.29525 (7)	0.33090 (6)	0.0532 (2)	
O4	0.6147 (3)	0.42483 (17)	0.34895 (19)	0.0640 (6)	
O3	0.3944 (3)	0.40232 (18)	0.32977 (18)	0.0637 (6)	
O2	0.9633 (2)	0.1945 (2)	0.58548 (19)	0.0614 (6)	
O1	0.7799 (3)	0.09198 (18)	0.57358 (19)	0.0621 (6)	
N1	0.9970 (3)	0.3094 (2)	0.5893 (2)	0.0568 (7)	
C5	0.6521 (2)	0.32586 (19)	0.57740 (17)	0.0341 (5)	
H5	0.644338	0.401627	0.582265	0.041*	
C11	0.5024 (3)	0.2451 (2)	0.31621 (16)	0.0363 (5)	
N2	0.2960 (3)	0.3124 (2)	0.3099 (2)	0.0592 (7)	
C14	0.5970 (3)	0.1692 (2)	0.31523 (17)	0.0361 (5)	
H14	0.563541	0.097687	0.305693	0.043*	
C3	0.8908 (3)	0.3632 (2)	0.58677 (19)	0.0418 (6)	
C16	0.8203 (3)	0.0795 (3)	0.3326 (2)	0.0465 (6)	
H16	0.79039	0.007312	0.331323	0.056*	
C15	0.7368 (3)	0.1771 (2)	0.32585 (17)	0.0364 (5)	
C6	0.5301 (2)	0.2673 (2)	0.56833 (16)	0.0324 (4)	
C9	0.3378 (3)	0.1419 (3)	0.5469 (2)	0.0550 (8)	
H9	0.277609	0.083236	0.537456	0.066*	
C10	0.5189 (3)	0.3637 (2)	0.3330 (2)	0.0465 (6)	
C12	0.3616 (3)	0.2241 (2)	0.30278 (18)	0.0416 (6)	
C7	0.4104 (3)	0.3206 (2)	0.56579 (19)	0.0418 (6)	
H7	0.403306	0.39636	0.570886	0.05*	
C2	0.7769 (2)	0.2927 (2)	0.58031 (17)	0.0355 (5)	
C8	0.3026 (3)	0.2476 (3)	0.5548 (2)	0.0519 (7)	
H8	0.216544	0.269318	0.553016	0.062*	
C1	0.8307 (3)	0.1822 (2)	0.5786 (2)	0.0443 (6)	
C13	0.2887 (3)	0.1175 (3)	0.2820 (2)	0.0532 (7)	
H13A	0.350705	0.060565	0.279027	0.08*	
H13B	0.253353	0.100902	0.332377	0.08*	
H13C	0.214967	0.121852	0.221289	0.08*	
C18	0.9717 (3)	0.2218 (3)	0.3399 (3)	0.0583 (8)	
H18	1.053929	0.253953	0.343547	0.07*	
C4	0.8928 (4)	0.4854 (3)	0.5881 (3)	0.0646 (9)	
H4A	0.805554	0.512482	0.585866	0.097*	
H4B	0.961917	0.510612	0.646195	0.097*	
H4C	0.912103	0.512135	0.533023	0.097*	
C17	0.9534 (3)	0.1117 (3)	0.3413 (3)	0.0566 (8)	
H17	1.022934	0.061462	0.347417	0.068*	

### Atomic displacement parameters (Å^2^)

**Table:**

	*U*^11^	*U*^22^	*U*^33^	*U*^12^	*U*^13^	*U*^23^
S1	0.0513 (4)	0.0345 (3)	0.0660 (5)	−0.0054 (3)	0.0238 (3)	−0.0015 (3)
S2	0.0498 (4)	0.0494 (4)	0.0636 (5)	−0.0118 (3)	0.0237 (3)	−0.0100 (3)
O4	0.0809 (17)	0.0329 (10)	0.0859 (16)	−0.0047 (11)	0.0387 (14)	−0.0056 (10)
O3	0.0730 (16)	0.0448 (12)	0.0793 (15)	0.0232 (11)	0.0341 (13)	0.0034 (11)
O2	0.0423 (11)	0.0631 (14)	0.0834 (16)	0.0140 (10)	0.0278 (11)	−0.0066 (12)
O1	0.0627 (14)	0.0389 (11)	0.0916 (17)	0.0077 (10)	0.0354 (13)	−0.0033 (11)
N1	0.0374 (12)	0.0693 (18)	0.0679 (16)	−0.0039 (12)	0.0237 (11)	−0.0109 (13)
C5	0.0345 (11)	0.0299 (10)	0.0394 (11)	0.0009 (9)	0.0147 (9)	−0.0022 (8)
C11	0.0417 (12)	0.0362 (12)	0.0331 (11)	0.0051 (10)	0.0157 (9)	0.0013 (9)
N2	0.0530 (15)	0.0618 (17)	0.0671 (16)	0.0191 (13)	0.0263 (13)	0.0006 (13)
C14	0.0374 (12)	0.0318 (11)	0.0422 (12)	−0.0015 (9)	0.0176 (10)	−0.0020 (9)
C3	0.0337 (12)	0.0526 (15)	0.0426 (12)	−0.0043 (11)	0.0176 (10)	−0.0049 (11)
C16	0.0339 (12)	0.0606 (17)	0.0494 (14)	−0.0057 (12)	0.0203 (11)	−0.0100 (12)
C15	0.0354 (12)	0.0373 (12)	0.0385 (11)	−0.0018 (9)	0.0152 (9)	−0.0033 (9)
C6	0.0322 (11)	0.0333 (11)	0.0336 (10)	−0.0009 (8)	0.0139 (8)	−0.0031 (8)
C9	0.0515 (17)	0.0628 (19)	0.0546 (16)	−0.0228 (15)	0.0234 (14)	−0.0033 (14)
C10	0.0633 (18)	0.0370 (13)	0.0442 (13)	0.0105 (12)	0.0250 (13)	0.0043 (10)
C12	0.0399 (13)	0.0478 (14)	0.0395 (12)	0.0097 (11)	0.0168 (10)	0.0036 (10)
C7	0.0351 (12)	0.0447 (14)	0.0470 (13)	0.0004 (10)	0.0159 (10)	−0.0069 (11)
C2	0.0322 (11)	0.0377 (12)	0.0384 (11)	−0.0002 (9)	0.0147 (9)	−0.0011 (9)
C8	0.0327 (12)	0.072 (2)	0.0532 (15)	−0.0035 (13)	0.0182 (11)	−0.0082 (14)
C1	0.0399 (14)	0.0447 (14)	0.0525 (14)	0.0071 (11)	0.0212 (11)	−0.0010 (11)
C13	0.0348 (13)	0.0605 (18)	0.0669 (18)	0.0018 (12)	0.0209 (13)	−0.0016 (14)
C18	0.0392 (15)	0.068 (2)	0.070 (2)	−0.0122 (14)	0.0218 (14)	−0.0102 (16)
C4	0.0546 (19)	0.0530 (18)	0.093 (2)	−0.0161 (15)	0.0339 (18)	−0.0061 (17)
C17	0.0416 (15)	0.064 (2)	0.0678 (19)	0.0066 (14)	0.0238 (14)	−0.0052 (15)

### Geometric parameters (Å, º)

**Table:**

S1—C9	1.695 (3)	C3—C4	1.487 (4)
S1—C6	1.725 (2)	C16—C17	1.410 (4)
S2—C18	1.691 (4)	C16—C15	1.457 (4)
S2—C15	1.727 (3)	C16—H16	0.93
O4—C10	1.204 (4)	C6—C7	1.399 (3)
O3—C10	1.370 (4)	C9—C8	1.354 (5)
O3—N2	1.460 (4)	C9—H9	0.93
O2—C1	1.365 (4)	C12—C13	1.481 (4)
O2—N1	1.437 (4)	C7—C8	1.400 (4)
O1—C1	1.210 (4)	C7—H7	0.93
N1—C3	1.280 (4)	C2—C1	1.460 (4)
C5—C2	1.354 (3)	C8—H8	0.93
C5—C6	1.427 (3)	C13—H13A	0.96
C5—H5	0.93	C13—H13B	0.96
C11—C14	1.357 (3)	C13—H13C	0.96
C11—C12	1.440 (4)	C18—C17	1.354 (5)
C11—C10	1.463 (4)	C18—H18	0.93
N2—C12	1.299 (4)	C4—H4A	0.96
C14—C15	1.420 (3)	C4—H4B	0.96
C14—H14	0.93	C4—H4C	0.96
C3—C2	1.444 (4)	C17—H17	0.93
			
C9—S1—C6	91.80 (14)	N2—C12—C11	112.7 (3)
C18—S2—C15	91.80 (15)	N2—C12—C13	119.5 (3)
C10—O3—N2	110.2 (2)	C11—C12—C13	127.8 (2)
C1—O2—N1	109.8 (2)	C6—C7—C8	112.8 (3)
C3—N1—O2	107.3 (2)	C6—C7—H7	123.6
C2—C5—C6	132.6 (2)	C8—C7—H7	123.6
C2—C5—H5	113.7	C5—C2—C3	126.2 (2)
C6—C5—H5	113.7	C5—C2—C1	130.3 (2)
C14—C11—C12	126.4 (2)	C3—C2—C1	103.5 (2)
C14—C11—C10	129.0 (3)	C9—C8—C7	112.3 (3)
C12—C11—C10	104.6 (2)	C9—C8—H8	123.9
C12—N2—O3	106.3 (3)	C7—C8—H8	123.9
C11—C14—C15	132.9 (2)	O1—C1—O2	121.1 (3)
C11—C14—H14	113.6	O1—C1—C2	132.2 (3)
C15—C14—H14	113.6	O2—C1—C2	106.6 (2)
N1—C3—C2	112.8 (3)	C12—C13—H13A	109.5
N1—C3—C4	120.2 (3)	C12—C13—H13B	109.5
C2—C3—C4	127.0 (3)	H13A—C13—H13B	109.5
C17—C16—C15	109.3 (3)	C12—C13—H13C	109.5
C17—C16—H16	125.4	H13A—C13—H13C	109.5
C15—C16—H16	125.4	H13B—C13—H13C	109.5
C14—C15—C16	121.5 (2)	C17—C18—S2	113.6 (2)
C14—C15—S2	127.6 (2)	C17—C18—H18	123.2
C16—C15—S2	110.91 (19)	S2—C18—H18	123.2
C7—C6—C5	122.3 (2)	C3—C4—H4A	109.5
C7—C6—S1	109.93 (19)	C3—C4—H4B	109.5
C5—C6—S1	127.71 (18)	H4A—C4—H4B	109.5
C8—C9—S1	113.2 (2)	C3—C4—H4C	109.5
C8—C9—H9	123.4	H4A—C4—H4C	109.5
S1—C9—H9	123.4	H4B—C4—H4C	109.5
O4—C10—O3	120.8 (3)	C18—C17—C16	114.4 (3)
O4—C10—C11	133.0 (3)	C18—C17—H17	122.8
O3—C10—C11	106.1 (3)	C16—C17—H17	122.8
			
C1—O2—N1—C3	1.2 (3)	O3—N2—C12—C13	−179.7 (2)
C10—O3—N2—C12	0.6 (3)	C14—C11—C12—N2	178.3 (3)
C12—C11—C14—C15	179.0 (3)	C10—C11—C12—N2	−0.4 (3)
C10—C11—C14—C15	−2.7 (5)	C14—C11—C12—C13	−2.2 (4)
O2—N1—C3—C2	−0.5 (3)	C10—C11—C12—C13	179.2 (3)
O2—N1—C3—C4	−179.0 (3)	C5—C6—C7—C8	178.9 (2)
C11—C14—C15—C16	173.0 (3)	S1—C6—C7—C8	0.4 (3)
C11—C14—C15—S2	−8.0 (4)	C6—C5—C2—C3	177.9 (2)
C17—C16—C15—C14	179.2 (2)	C6—C5—C2—C1	−2.6 (5)
C17—C16—C15—S2	0.0 (3)	N1—C3—C2—C5	179.3 (3)
C18—S2—C15—C14	−178.4 (2)	C4—C3—C2—C5	−2.3 (5)
C18—S2—C15—C16	0.7 (2)	N1—C3—C2—C1	−0.3 (3)
C2—C5—C6—C7	−179.2 (3)	C4—C3—C2—C1	178.1 (3)
C2—C5—C6—S1	−0.9 (4)	S1—C9—C8—C7	1.8 (4)
C9—S1—C6—C7	0.5 (2)	C6—C7—C8—C9	−1.4 (4)
C9—S1—C6—C5	−177.9 (2)	N1—O2—C1—O1	179.3 (3)
C6—S1—C9—C8	−1.3 (3)	N1—O2—C1—C2	−1.4 (3)
N2—O3—C10—O4	−179.7 (3)	C5—C2—C1—O1	0.6 (6)
N2—O3—C10—C11	−0.8 (3)	C3—C2—C1—O1	−179.8 (3)
C14—C11—C10—O4	0.8 (5)	C5—C2—C1—O2	−178.6 (3)
C12—C11—C10—O4	179.4 (3)	C3—C2—C1—O2	1.0 (3)
C14—C11—C10—O3	−177.9 (2)	C15—S2—C18—C17	−1.2 (3)
C12—C11—C10—O3	0.7 (3)	S2—C18—C17—C16	1.5 (4)
O3—N2—C12—C11	−0.1 (3)	C15—C16—C17—C18	−0.9 (4)

### Hydrogen-bond geometry (Å, º)

**Table:**

*D*—H···*A*	*D*—H	H···*A*	*D*···*A*	*D*—H···*A*
C7—H7···O4^i^	0.93	2.51	3.387 (3)	156
C8—H8···N1^ii^	0.93	2.58	3.491 (5)	166
C13—H13c···N1^iii^	0.96	2.57	3.487 (4)	160

Symmetry codes: (i) −*x*+1, −*y*+1, −*z*+1; (ii) *x*−1, *y*, *z*; (iii) *x*−1, −*y*+1/2, *z*−1/2.
